# Enhancing Surface Termination and Stability of Hybrid Halide Perovskites via Phosphonic Acid Passivation

**DOI:** 10.1021/acsomega.5c12245

**Published:** 2026-04-28

**Authors:** Israel C. Ribeiro, Iván Ornelas-Cruz, Felipe D. Picoli, Luiz N. Oliveira, Matheus P. Lima, Ana Flávia Nogueira, Juarez L. F. Da Silva

**Affiliations:** † São Carlos Institute of Chemistry, University of São Paulo, Av. Trabalhador São-Carlense 400, São Carlos, São Paulo 13560-970, Brazil; ‡ São Carlos Institute of Physics, University of São Paulo, Av. Trabalhador São-Carlense 400, São Carlos, São Paulo 13560-970, Brazil; § Department of Physics, Federal University of São Carlos, São Carlos, São Paulo 13565-905, Brazil; ∥ Laboratório de Nanotecnologia e Energia Solar, Chemistry Institute, University of Campinas, Campinas, São Paulo 13083-970, Brazil

## Abstract

Surface passivation at hybrid halide perovskite interfaces is critical for suppressing nonradiative recombination and improving operational stability, but the molecular-scale mechanisms remain incompletely understood. Here, we use density functional theory to investigate the adsorption of phenylphosphonic acid (PPA) and 2-carboxyethylphosphonic acid (CEPA) on representative surfaces CH_3_NH_3_PbI_3_ (MAPbI_3_) using periodic slab models. Both acids bind exergonically to all terminations considered, with CEPA consistently exhibiting stronger adsorption than that of PPA as a result of additional hydrogen bonding enabled by its carboxyl group. Adsorption becomes more favorable with increasing exposure to the undercoordinated surface Pb sites, consistent with Pb–O coordination as the dominant anchoring motif. Charge-density-difference analysis and atom-resolved charge partitioning reveal substantial interfacial charge redistribution, while local density-of-states calculations show adsorption-induced hybridization near the valence-band edge without introducing localized midgap states. CEPA also produces larger adsorption-induced interfacial dipoles, raising the (VBM-referenced) work-function upper bound to values above 5.9 eV. In general, these results support phosphonic acids—particularly CEPA—as effective molecular passivators that stabilize MAPbI_3_ surfaces and tune interfacial energetics relevant to perovskite optoelectronic devices.

## Graphical abstract

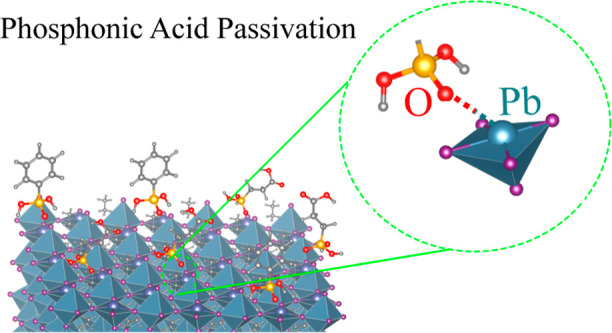

## 1 Introduction

Hybrid organic–inorganic halide perovskites have emerged as leading photovoltaic semiconductors because they combine strong optical absorption, long carrier diffusion lengths, balanced ambipolar transport, and low-temperature solution deposition.^1–3^ Although state-of-the-art lab-scale devices now achieve power-conversion efficiencies (PCEs) approaching 27%,^4^ this performance still falls short of the Shockley–Queisser single-junction limit of about 33%.^5^ Bridging this gap has motivated intensive efforts to optimize processing and tune the perovskite crystal structure and electronic properties.

A major challenge is mitigating defects on the surfaces and grain boundaries, which act as recombination centers that limit carrier lifetimes and device performance. Strategies including surface functionalization, additive engineering, and controlled doping can improve efficiency and operational stability.^6,7^ Nevertheless, a molecular understanding of how specific chemical treatments passivate perovskite surfaces remains essential for rational and predictive interface design.

The intrinsic instability of organic and halide components in perovskite films can lead to volatilization under thermal stress or illumination, generating undercoordinated centers Pb^2+^ and other unsaturated surface sites.^8,9^ Due to the soft ionic lattice, such defects easily create charged trap states that capture photogenerated carriers and impede charge transport. Interfacial defects are particularly detrimental because they act as nonradiative recombination centers, reducing the open-circuit voltage (*V*_oc_) and the fill factor.^10^ These defective regions can also facilitate the ingress of moisture and oxygen, accelerating degradation. Therefore, controlling and passivating the interfaces between the perovskite absorber and the charge-transport layers (CTLs) is critical to improving both operational stability and power-conversion efficiency.^9^

Mitigating surface defects is therefore essential for improving the performance and stability of perovskite thin films. Among passivation strategies, Lewis-base additives are particularly effective, and phosphoryl-functionalized molecules have attracted attention.^9,11^ The P=O group coordinates strongly to Pb^2+^, often producing stronger coordination than other neutral functionalities such as C=O, S=O, N=O, or As=O. As an example, triphenylphosphine oxide (TPPO) can suppress trap-state density, enhance photoluminescence, and improve long-term device stability.

Aalbers et al.^12^ examined how substrate functionalization mitigates nonradiative recombination in perovskite films. Functionalization of glass and indium tin oxide with phosphonic acids, including phenylphosphonic acid (PPA), improved film quality, and increased quasi-Fermi level splitting (QFLS), consistent with reduced nonradiative losses that limit *V*_oc_ in perovskite solar cells (PSCs). Their results indicate that substrate modification can reduce interfacial recombination even on noncharge-selective contacts, providing a useful platform for analyzing voltage losses and guiding defect-management strategies across perovskite compositions.

Experimental studies further support the passivation activity of PPA. For example, Zhou et al.^13^ reported that the introduction of PPA at the SnO_2_/MAPbI_3_ interface (where MA = CH_3_NH_3_^+^) improved device performance, which they attributed to reduced interfacial recombination and improved energy-level alignment. Furthermore, Singh et al.^14^ also reported improved performance using PPA-based passivators, highlighting the greater potential of phosphonic-acid chemistry for defect management in PSCs.

Here, we use density functional theory (DFT) to examine the adsorption and passivation mechanisms of phenylphosphonic acid (PPA, C_6_H_7_O_3_P) and 2-carboxyethylphosphonic acid (CEPA, C_3_H_7_O_5_P) on MAPbI_3_ surfaces with MAI-, PbI_2_-, and mixed terminations. Across the terminations considered, CEPA binds more strongly than PPA, consistent with additional hydrogen bonding enabled by its carboxyl group. Passivation is most pronounced on Pb-rich terminations, where Pb–O coordination provides the dominant anchoring motif. Charge-density redistribution and local density-of-states analyses indicate adsorption-induced changes in band-edge hybridization and work-function shifts, with CEPA producing larger changes and thus stronger modulation of interfacial energetics. Overall, these results support phosphonic acids—particularly CEPA—as effective molecular passivators for improving the stability and optoelectronic properties of perovskite interfaces.

## 2 Theoretical Approach and Computational Details

### 2.1 Total Energy Calculations

All electronic structure calculations were performed within the DFT framework using the semilocal Perdew–Burke–Ernzerhof (PBE) exchange–correlation energy functional,^15^ as implemented in the Vienna Ab initio Simulation Package (VASP, version 5.4.4).^16,17^ In VASP, the Kohn–Sham orbitals are represented in a plane-wave basis set, and core–valence interactions are described using the projector-augmented-wave (PAW) method.^18,19^ Long-range van der Waals interactions, which are not adequately described by semilocal functionals in molecule–surface adsorption systems,^20,21^ were included via Grimme’s D3 dispersion correction,^22^ in all calculations to improve the description of phosphonic-acid adsorption on selected MAPbI_3_ surfaces.

To capture adsorbate–adsorbate and adsorbate–substrate effects, we relaxed the in-plane lattice parameters (*a*_0_ and *b*_0_) and atomic positions for all slab models. Equilibrium geometries were obtained by minimizing the in-plane (*xy*) components of the stress tensor while relaxing atomic positions until the residual forces in all directions (*x*, *y*, and *z*) were below the chosen convergence thresholds.^23^ Calculations used a plane-wave basis set with an energy cutoff of 1.50 × ENMAX_max_, where ENMAX_max_ is the largest recommended cutoff among the selected PAW projectors for the constituent elements (631.353 eV).

For all subsequent calculations, including interaction and adsorption energies, the density of states (DOS), the work function, and net atomic charges computed using the density-derived electrostatic and chemical (DDEC) scheme,^24,25^ we used a uniform plane-wave kinetic energy cutoff of 473.515 eV. Brillouin-zone integrations employed *k*-point meshes of 2 × 2 × 1 for slab geometry optimizations and 4 × 4 × 1 for subsequent property calculations. Structural optimizations were deemed to converge when the maximum residual force in any atom was below 2.50 × 10^–2^ eV/Å and electronic self-consistency was reached with a total-energy change below 10^–5^ eV. For all subsequent property calculations, the electronic convergence criterion was set to 10^–6^ eV.

### 2.2 Surface Models and Adsorption Configurations

#### 2.2.1 MAI Surface Models

To model the two-dimensional (100) surfaces of MAPbI_3_, we constructed slab models from a three-dimensional orthorhombic 2 × 2 × 2 supercell. To avoid spurious interactions between periodic images, the slabs were separated by a vacuum region of 15 Å. The resulting structures are consistent with the chemical formula MA_5_Pb_4_I_13_, in which MA cations compensate the negative surface charge. According to the electron count rule, four MA cations are required on each slab face (top and bottom) to ensure full surface passivation. The thickness of the slab was defined by the number of ${\left[{PbI}_{6}\right]}^{4-}$ octahedral layers, denoted by *n*. In this study, we used slabs with *n* = 4 layers (a four-layer, 4*L*, arrangement). Consequently, the final model, MA_20_Pb_16_I_52_ (*n* = 4, 4*L*), corresponds to a surface terminated with 4MAI (Figure 1).^26^

**Figure 1:**
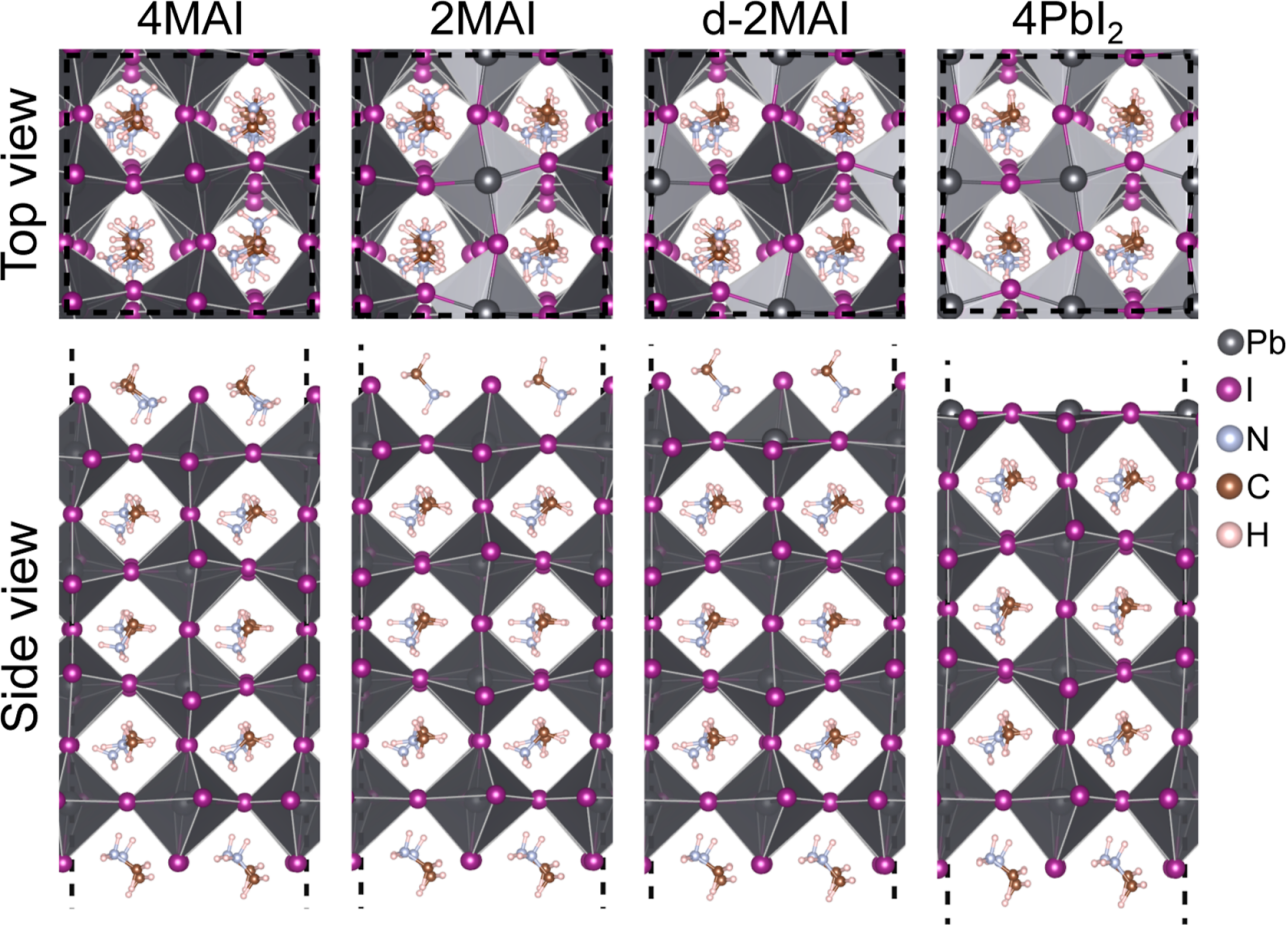
Top and side views of the 4MAI, 2MAI, d-2MAI, and 4PbI_2_ structural models are shown. In the 4MAI configuration, the surfaces are fully passivated by MA species, with four MA cations per crystallographic face. The 2MAI and d-2MAI models exhibit partial passivation, with two MA cations per face; the d-2MAI model additionally incorporates a single MA vacancy on one side of the structure. The 4PbI_2_ model, in turn, presents surfaces exposing undercoordinated Pb atoms.

#### 2.2.2 Pb-Rich Surface Models

To assess how the degree of surface exposure Pb affects the bonding of Pb–O, we constructed additional slab models derived from 4MAI. Three models were generated by selectively removing MA cations and iodide anions from the top surface to increase the density of the undercoordinated surface Pb sites while maintaining overall charge neutrality. In the fully exposed 4PbI_2_ model, all top-layer MA and iodide species were removed, leaving all surface lead atoms undercoordinated. To represent intermediate vacancy concentrations, we built two half-vacancy models: in 2MAI, the vacancies expose the lead atoms aligned along the *a*_0_ axis, whereas in d-2MAI, the vacancies expose the lead atoms oriented diagonally with respect to *a*_0_. This controlled variation in vacancy density and symmetry (Figure 1) enables a detailed analysis of how lead–phosphonate interactions depend on surface structure.

#### 2.2.3 Molecular Adsorption

Guided by the findings of Aalbers et al.,^12^ we selected phenylphosphonic acid (PPA, C_6_H_7_O_3_P) and 2-carboxyethylphosphonic acid (CEPA, C_3_H_7_O_5_P) (Figure 2) as representative phosphonic-acid passivators. This choice reflects their ability to anchor to oxide substrates through phosphonate groups and to modulate interfacial energetics relevant to nonradiative recombination. To evaluate adsorption trends between terminations, PPA and CEPA were adsorbed in the 4MAI, 2MAI, d-2MAI, and 4PbI_2_ slab models. The number of adsorbates was chosen to match the surface stoichiometry of each model: four molecules in 4MAI and 4PbI_2_, and two molecules in 2MAI and d-2MAI. For surfaces exposing undercoordinated Pb atoms, the phosphonic groups were initially oriented to promote Pb–O coordination, producing chemically reasonable binding motifs consistent with experimental observations.

**Figure 2:**
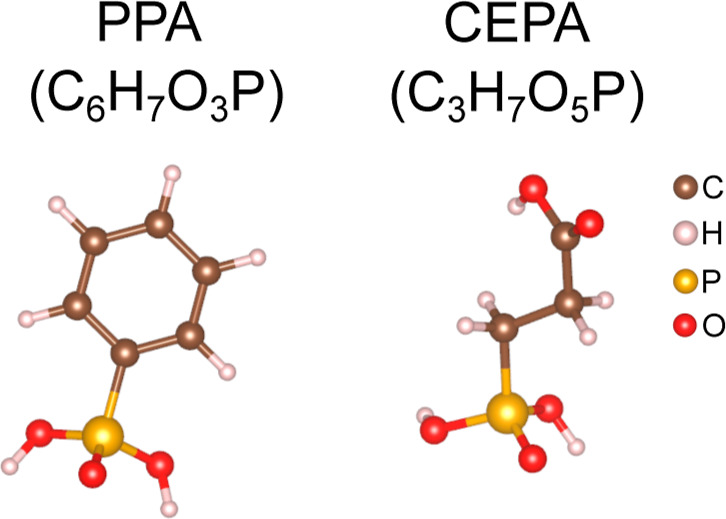
Molecular representation of the phenylphosphonic acid and 2-carboxyethylphosphonic acid.

## 3 Results and Discussion

Initial geometry optimizations were used to identify the lowest-energy surface models (4MAI, 2MAI, d-2MAI, and 4PbI_2_; Figure 1). We then analyzed PPA and CEPA adsorption to obtain atomistic insight into Pb–O coordination and to quantify the resulting structural, energetic, and electronic changes using a consistent set of descriptors. The following sections summarize the main findings.

### 3.1 Surface Characterization of Perovskite Models

To characterize the 4MAI, 2MAI, d-2MAI, and 4PbI_2_ surface models, we evaluated a set of geometric and electronic descriptors. These include the equilibrium in-plane lattice parameters (*a*_0_ and *b*_0_), the average short and long Pb–I bond lengths within the PbI_6_ octahedra (S-*d*_av_^Pb‑I^ and L-*d*_av_^Pb‑I^), and the average effective coordination number of lead (ECN_av_^Pb^). Octahedral distortions were further quantified using the average Pb–I–Pb and I–Pb–I bond angles (θ_av_^PbIPb^ and θ_av_^IPbI^). In addition, we computed the electronic band gap (*E*_g_) and work function (Φ). All values are summarized in Table 1.

**Table 1:** Structural Descriptors for the 4MAI, 2MAI, D-2MAI and 4PbI_2_ Surface Models and for the 3D Bulk MAPbI_3_: Lattice Parameters (*a*_0_ and *b*_0_), the Average Bond Distance of the Short (S-*d*_av_^Pb‑I^) and Long (L-*d*_av_^Pb‑I^) Bonds in the PbI_6_-Octahedra, Effective Coordination Number of Lead (ECN_av_^Pb^), the Average Bond Angles of Pb–I–Pb (θ_av_^PbIPb^) and I–Pb–I (θ_av_^IPbI^)­^a^

surface models	*a*_0_ (Å)	*b*_0_ (Å)	<keep-together > S-*d*_av_^Pb–I^</keep-together> (Å)	<keep-together > L-*d*_av_^Pb–I^</keep-together> (Å)	ECN_av_^Pb^ (NNN)	θ_av_^PbIPb^ (°)	θ_av_^IPbI^ (°)	*E*_g_ (eV)	Φ (eV)
4MAI	12.24	12.71	3.13	3.21	5.89	154.25	175.56	1.60	4.89
2MAI	12.27	12.73	3.03	3.21	5.27	159.30	170.23	1.34	5.21
d-2MAI	12.29	12.71	3.00	3.21	5.31	157.98	172.60	1.44	5.27
4PbI_2_	12.32	12.78	3.00	3.16	4.74	160.18	168.93	1.03	5.67
3D bulk MAPbI_3_	12.22	12.26	3.16	3.16	5.99	168.56	171.83	1.78	

a In addition, the electronic descriptors: electronic energy band gap (*E*_g_) and work function (Φ).

#### 3.1.1 Structural Descriptors of Surface Models

For 3D bulk MAPbI_3_, we obtained *a*_0_ = 12.22 Å and *b*_0_ = 12.26 Å, which differ by 0.04 Å. The four surface terminations have similar equilibrium lattice parameters, with *a*_0_ = 12.24–12.32 Å and *b*_0_ = 12.71–12.78 Å (average *a*_0_–*b*_0_ difference of 0.47 Å), indicating lattice distortions induced by the surface regions (top and bottom of the slab). The magnitude of *a*_0_–*b*_0_ further indicates that the PbI_6_ octahedral framework is distorted, consistent with the effective coordination numbers and the average bond angles in Table 1.

Analysis of the Pb–I bonding environment reveals systematic trends in PbI_6_ octahedral distortion as a function of surface MAI content. The slabs exhibit two average Pb–I bond lengths, S-*d*_av_^Pb‑I^ (short) and L-*d*_av_^Pb‑I^ (long), reflecting local structural asymmetry. The 4MAI model has the longest average Pb–I bonds and the highest lead coordination (ECN_av_^Pb^ = 5.89 NNN), consistent with a comparatively less distorted PbI_6_ octahedral framework. In contrast, 4PbI_2_ and d-2MAI show shorter average Pb–I bonds (down to 3.00 Å) and lower ECN_av_^Pb^ (down to 4.74 NNN in the upper layer of 4PbI_2_), indicating reduced Pb^2+^ and stronger surface distortions that can lower surface energy.

Furthermore, the Pb–I–Pb bond angles deviate substantially from the ideal linear value of 180° for all surface terminations, indicating pronounced octahedral tilting induced by surface formation. The deviation is most pronounced for 4MAI, where θ_av_^PbIPb^ decreases to 154.25°, corresponding to a distortion of nearly 26°. In contrast, the fully inorganic 4PbI_2_ termination increases θ_av_^PbIPb^ to 160.18°, reflecting reduced tilting relative to 4MAI; nevertheless, the angle remains markedly nonlinear, with a residual deviation of approximately 20°. Consequently, removing the organic termination only partially alleviates the octahedral tilting, and pronounced octahedral rotations persist even for the purely inorganic surface.

#### 3.1.2 Valence States in Surface Models

The local density of states (LDOS) for the 4MAI, 2MAI, d-2MAI, and 4PbI_2_ surfaces (Figure 3) shows that all terminations retain the semiconducting character of bulk MAPbI_3_, with band edges dominated by the inorganic PbI_6_ framework. The valence-band maximum (VBM) is dominated by I *p* states with minor Pb *s* hybridization, arising from antibonding I 5p–Pb 6s interactions. The conduction-band minimum (CBM) is largely derived from Pb p states with smaller I contributions, consistent with antibonding states centered on Pb that govern electron transport. The band gap systematically decreases from the MAI-terminated 4MAI surface to the Pb-rich 4PbI_2_ termination, reflecting progressive band-edge reconstruction. No localized midgap states appear in any model, indicating that surface formation modifies extended states rather than introducing defect levels.

**Figure 3:**
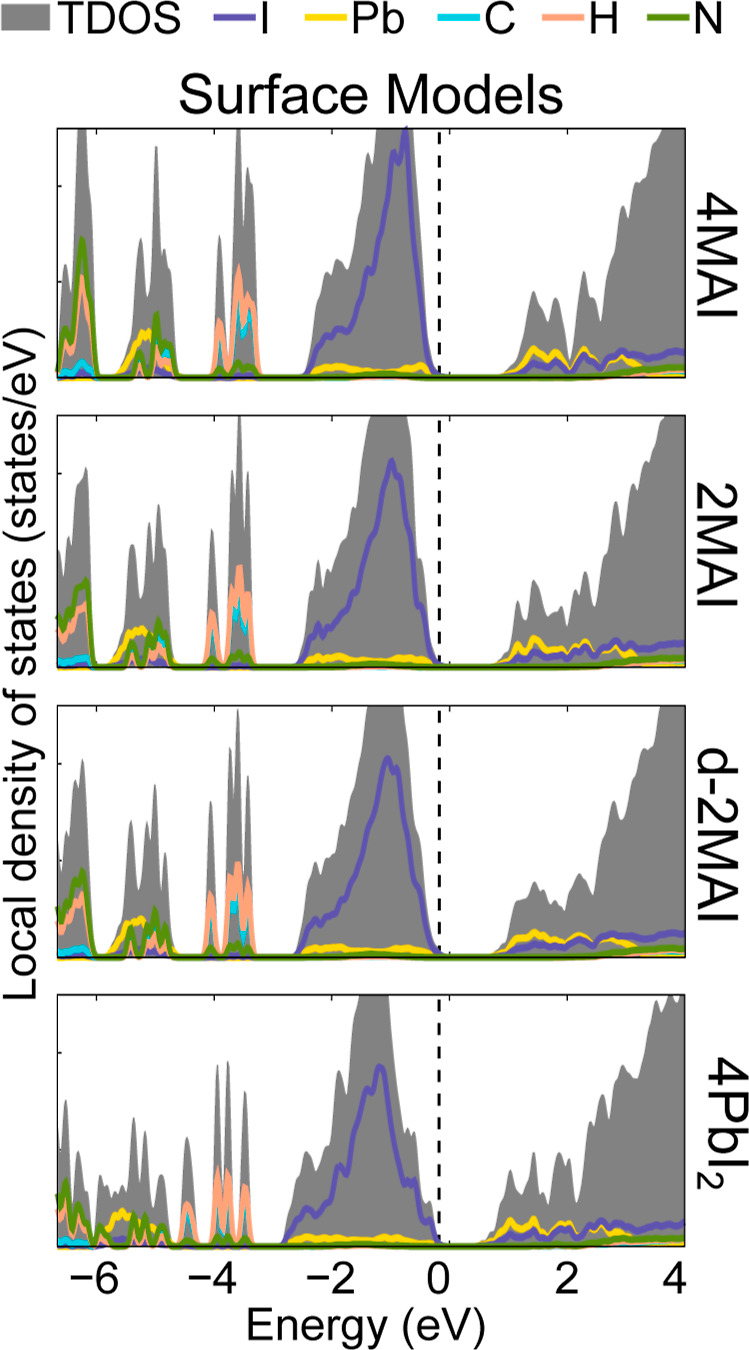
Local density of states for the investigated perovskite surface models, namely 4MAI, 2MAI, d-2MAI, and 4PbI_2_. The energy scale is referenced such that the valence band maximum is fixed at 0 eV (indicated by the vertical dashed line).

The evolution of the band gap (*E*_g_) follows the progressive reconstruction of the PbI_6_ octahedra summarized in Table 1. As MAI coverage decreases, the surface Pb becomes increasingly undercoordinated, with short contracted Pb–I bonds and larger deviations of Pb–I–Pb angles from linearity, indicative of enhanced tilting and local asymmetry. These distortions are correlated with the monotonic decrease in *E*_g_, from 1.60 eV for 4MAI to 1.03 eV for 4PbI_2_. Increased tilt and bond contraction strengthen orbital overlap in antibonding states Pb–I near the band edges, raising the VBM and lowering the CBM. Thus, surface distortion and Pb undercoordination narrow the gap via band-edge reconstruction rather than by creating defect states; octahedral tilting and loss of coordination are the main drivers of the reduction of the gap at these surfaces.

#### 3.1.3 Work Function of the Surface Models

The work function (Φ) is the energy required to remove an electron from the material at the Fermi level and place it in the vacuum region (at the vacuum level). Thus, it is given by

1 $$\Phi =V_{es}\left(r_{vac}\right)-E_{Fermi}$$

where *V*_es_(**r**_vac_) is the electrostatic potential in the middle of the vacuum region of the slab and *E*_Fermi_ is the Fermi energy. For metals, Φ coincides with the ionization energy (IE) and the electron affinity (EA), since the occupied and unoccupied states are not separated by a band gap. For a nondegenerate semiconductor, Φ lies between IE and EA because *E*_Fermi_ falls within the band gap and depends on temperature, carrier density, and doping. In MAPbI_3_, however, the Fermi level is not necessarily located near midgap, as the material exhibits n-type behavior, with *E*_Fermi_ shifted closer to the CBM even for different substrates.^27^

In this work, we estimate Φ using its upper bound (i.e., the ionization energy), taking the valence band maximum (VBM) as the reference energy

2 $$\Phi \approx V_{es}\left(r_{vac}\right)-E_{VBM}$$

This choice is further justified by the well-known band gap underestimate arising from the difference between Kohn–Sham VBM and CBM energies; in MAPbI_3_ systems, the apparent accuracy at this level of theory is attributed to the cancelation of errors.^28^ Throughout the manuscript, we refer to this VBM-referenced quantity as Φ for simplicity; it corresponds to the slab ionization potential referenced to vacuum and provides an upper bound to the work function of a semiconductor. The resulting values strongly depend on the surface composition (Table 1). Among the surfaces studied, 4MAI has the lowest Φ, 4.89 eV, and 4PbI_2_ the highest 5.67 eV, while 2MAI and d-2MAI exhibit intermediate values of 5.21 and 5.27 eV, respectively.

A lower Φ (or equivalently a smaller ionization energy in this approximation) can reduce energetic barriers for electron extraction at perovskite/electron–transport-layer interfaces, facilitating charge transfer and mitigating interfacial recombination.^29,30^ Therefore, MAI-rich surfaces, with lower Φ, are expected to favor charge transfer processes (electron injection or hole extraction, depending on device architecture). In contrast, the higher Φ of the PbI_2_-rich surface reflects a stronger surface dipole and/or a more polarized surface electrostatic environment characteristic of lead-rich terminations. Because global charge neutrality is preserved upon removing neutral pairs MAI, the observed variations in Φ are mainly driven by termination-dependent surface stoichiometry and dipole formation rather than by bulk-like *n* or *p*-type doping.

### 3.2 Energetic Stability of Surface-Acids Binding

To quantify the thermodynamic stability of the adsorbed systems (Figure 4), we calculated the adsorption energy (*E*_ad_) using eq 3

3 $$E_{ad}=E_{tot}^{acids/slab}-E_{tot}^{slabrelaxed}-E_{tot}^{acidsfree}$$

where *E*_tot_^acids/slab^ is the total energy of the slab with adsorbed phosphonic acids, $E_{tot}^{slabrelaxed}$ is the total energy of the optimized perovskite slab without adsorbates and $E_{tot}^{acidsfree}$ is the total energy of the isolated PPA and CEPA molecules. We also compute the interaction energy (*E*_int_), defined in eq 4, to quantify the interaction strength between the phosphonic acids and the perovskite surface. Using $E_{tot}^{slabfrozen}$ and $E_{tot}^{acidsfrozen}$ , i.e., the total energies of the slab and the acids frozen in their optimized adsorption geometries, we remove the contributions of the isolated components

4 $$E_{\mathrm{int}}=E_{tot}^{acids/slab}-E_{tot}^{slabfrozen}-E_{tot}^{acidsfrozen}$$

Here, the adsorption energy (*E*_ad_) captures the overall stabilization when a gas-phase molecule binds to the surface, including molecular and surface rearrangements. In contrast, the interaction energy (*E*_int_) reflects the intrinsic molecule–surface interaction in the adsorbed state by excluding these relaxation effects. For all systems studied, both *E*_ad_ and *E*_int_ are negative, indicating a thermodynamically favorable binding of acids to the surfaces.

**Figure 4:**
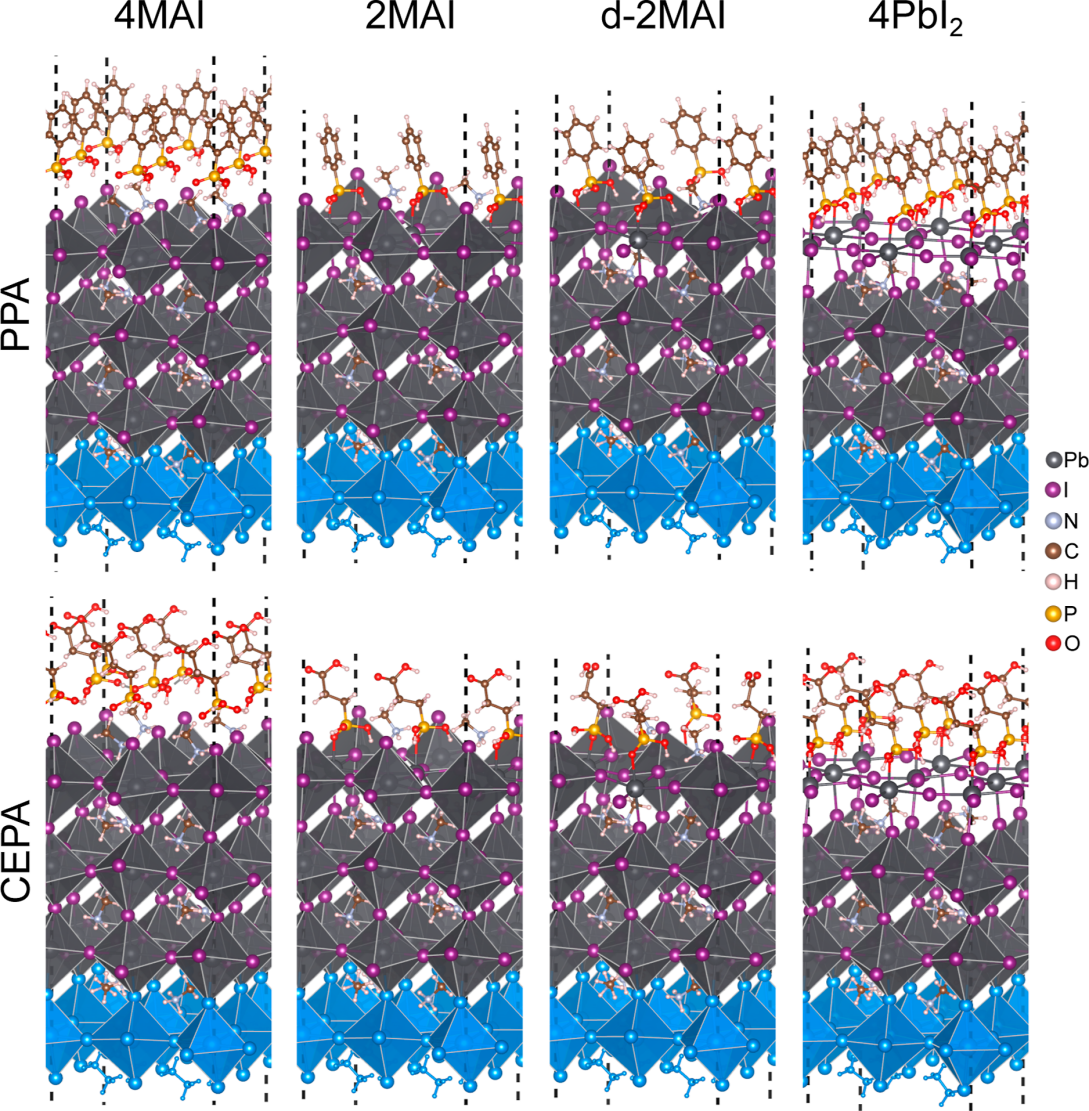
Side views for the lowest energy 4MAI, 2MAI, d-2MAI, and 4PbI_2_ models considering the adsorption of PPA and CEPA acids and an rotation angle of 10° for better visualization. The dashed line represents the unit cell and blue octahedra indicate the frozen atoms in the slab model (surface bottom).

The adsorption energies are strongly dependent on the molecular structure of the R group in R–PO­(OH)_2_. As shown in Figure 5, CEPA exhibits more negative *E*_ad_ values than PPA in all surface models. The largest relative difference in *E*_ad_ (∼37%) occurs on the 4MAI surface. The carboxyl group (−COOH) in CEPA strengthens adsorption by enabling additional intermolecular interactions, particularly hydrogen bonding. However, both acids show similar trends in *E*_ad_ as a function of the perovskite surface model.

**Figure 5:**
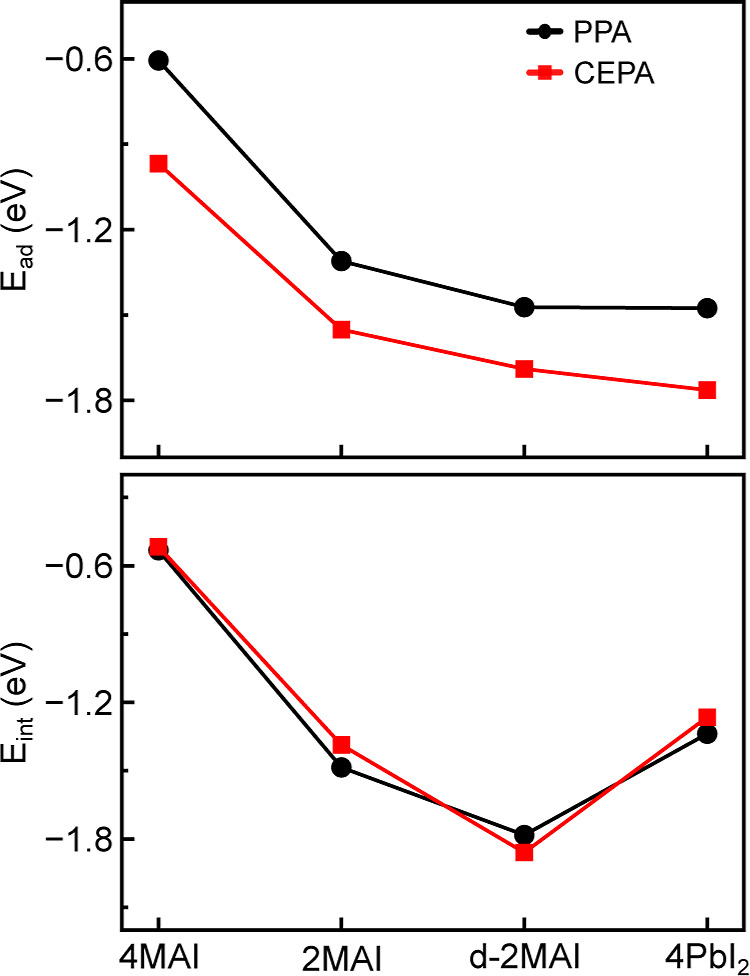
Top panel shows the adsorption (*E*_ad_), and bottom panel shows the interaction energies (*E*_int_) of PPA and CEPA on perovskite surface models: 4MAI, 2MAI, d-2MAI and 4PbI_2_.

The stability of adsorption is additionally modulated by the surface symmetry of the perovskite. In agreement with previous reports,^12^ Pb–O interactions constitute the primary stabilizing factor for the adsorbed acids. Consequently, adsorption stability follows the sequence 4MAI < 2MAI < d-2MAI < 4PbI_2_, with the fully Pb-terminated 4PbI_2_ surface exhibiting the strongest adsorption. In particular, the d-2MAI model displays adsorption energies comparable to those of 4PbI_2_, underscoring the contribution of intermolecular interactions between the acids and the surface moieties.

In contrast, acid–surface interaction energies show much smaller percentage deviations. After adsorption, both PPA and CEPA yield similar *E*_int_ values (Figure 5). Across all surface models, CEPA generally gives more exergonic interaction energies than PPA, except for d-2MAI, where the ordering is reversed. This inversion reflects the distinct interaction environment of d-2MAI, likely due to stronger intermolecular interactions between the acids and MA species on the surface. Highlights how a dense hydrogen-bonding network or cooperative adsorption can markedly enhance binding, depending on the molecular arrangement and surface termination.

### 3.3 Effective Charges

To clarify how effective charges at different perovskite surface sites evolve after acid passivation, we used the DDEC method,^24,25^ to compute the effective charges resolved by atoms (Table 2). Oxygen atoms directly bound to Pb (*Q*_eff_^O*^) become more negative, accompanied by an increase in the positive charge on bonded Pb atoms, consistent with strong Pb–O interactions that anchor acids at the surface. For both acids, passivation also increases the positive character of phosphorus, indicating charge transfer toward the coordinating oxygens. In contrast, iodine sites change only slightly, suggesting that halide sublattice is weakly perturbed by adsorption. The MA cations similarly retain nearly constant charges, consistent with a spectator role in surface binding.

**Table 2:** Effective DDEC Charges Obtained with the PPA and CEPA Acids on the 4MAI, 2MAI, D-2MAI, and 4PbI_2_ Surface models^a^

acid	surface	*Q*_eff_^O*^ (*e*)	*Q*_eff_^Pb*^ (*e*)	*Q*_eff_^O^ (*e*)	*Q*_eff_^Pb^ (*e*)	*Q*_eff_^P^ (*e*)	*Q*_eff_^I^ (*e*)	*Q*_eff_^MA^ (*e*)
PPA	4MAI			–0.66	0.72	1.24	–0.47	0.66
	2MAI	–0.72	0.82	–0.65	0.74	1.27	–0.46	0.66
	d-2MAI	–0.72	0.83	–0.65	0.74	1.27	–0.47	0.65
	4PbI_2_	–0.71	0.89	–0.64	0.77	1.26	–0.47	0.67
CEPA	4MAI			–0.59	0.72	1.31	–0.46	0.67
	2MAI	–0.68	0.84	–0.59	0.74	1.32	–0.46	0.63
	d-2MAI	–0.71	0.85	–0.58	0.74	1.32	–0.46	0.65
	4PbI_2_	–0.72	0.86	–0.59	0.77	1.33	–0.48	0.66

a Reported values correspond to the average charges on oxygen (*Q*_eff_^O^), lead (*Q*_eff_^Pb^), phosphorus (*Q*_eff_^P^), iodine (*Q*_eff_^I^), and methylammonium (*Q*_eff_^MA^) sites. The superscript * indicates atoms involved in the Pb–O bond.

A clear distinction emerges between PPA and CEPA. In CEPA, oxygen atoms distal to Pb carry slightly less negative charge, while the phosphorus center becomes more positively charged, indicating stronger internal polarization of the phosphonic-acid group. This enhanced polarization strengthens the donor–acceptor character of the Pb–O bonds, consistent with the higher binding affinity of CEPA’s and improved suppression of surface trap states. In general, these results show that the electronic stabilization from acid passivation is primarily due to the redistribution of charges within the motif Pb–O–P, while the inorganic framework and organic spacer units remain largely unchanged. This selective modulation of interfacial charge density provides a microscopic basis for improved electronic passivation and stability in phosphonic-acid-treated perovskite thin films.

### 3.4 Charge Reorganization Mapped via Electron Density Difference

To elucidate the key features of molecular adsorption on perovskite thin-film surfaces, we analyzed the electron density difference (EDD) as a descriptor of charge redistribution upon interface formation. The EDD was computed as

5 $$\Delta \rho =\rho^{acids/slab}-\rho^{acids}-\rho^{slab}$$

Here, ρ^acids/slab^ is the electron density of the optimized acid–surface adsorption complex, while ρ^acids^ and ρ^slab^ are the electron densities of the isolated acids and the slab, respectively, evaluated in frozen geometries extracted from the optimized adsorption configuration. The resulting EDD maps (Figure 6) reveal how charge reorganization depends on the degree of surface exposure Pb and the chemical structure of the phosphonic-acid group.

**Figure 6:**
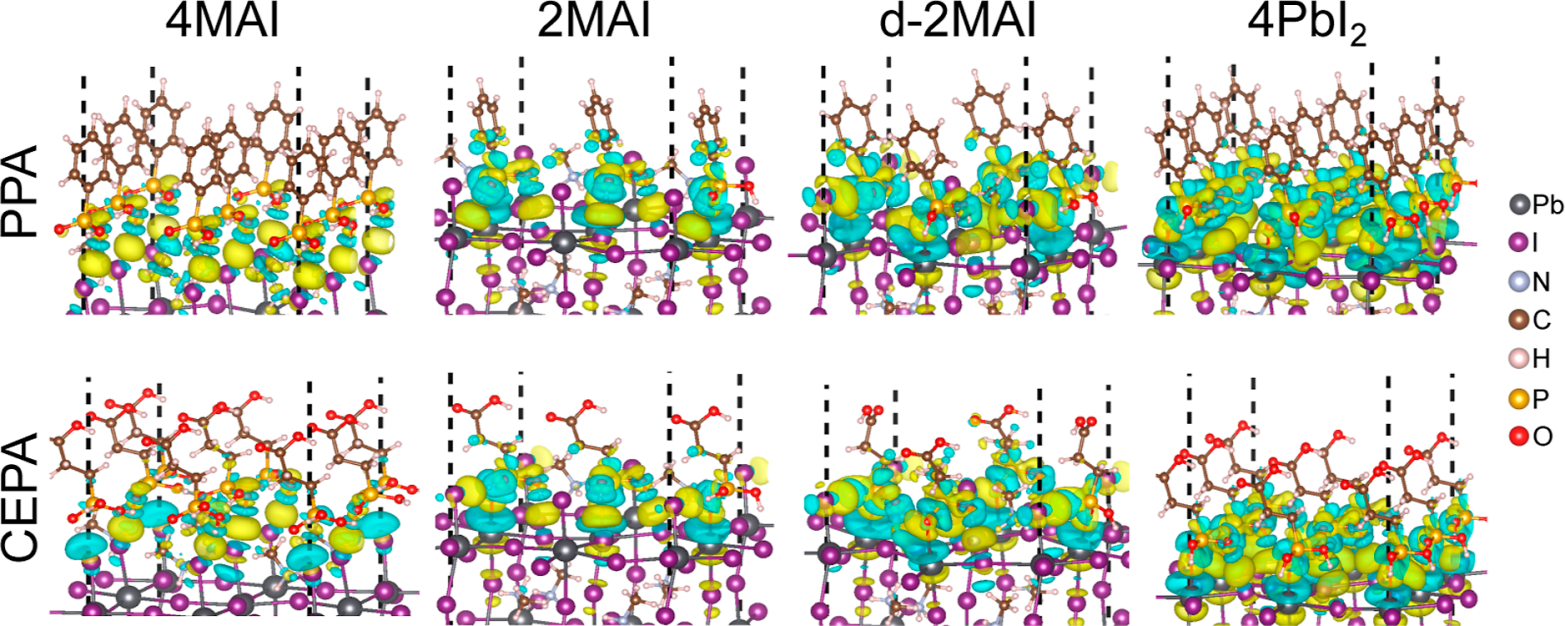
Electron density difference isosurfaces (0.001 0 bohr^–3^) for the PPA and CEPA on perovskite surface models: 4MAI, 2MAI, d-2MAI and 4PbI_2_. The yellow and blue regions indicate the accumulation and depletion of charge, respectively.

For both PPA and CEPA, the EDD maps show qualitatively similar charge-accumulation and charge-depletion patterns, indicating charge transfer upon adsorption. PPA adsorption produces slightly more intense isosurfaces, suggesting a stronger electronic redistribution at the interface. For MA-terminated surfaces, charge redistribution is weaker than for Pb-exposed terminations, as reflected by smaller isosurfaces. In 4MAI, the EDD maps show charge depletion near a methyl hydrogen of the MA cations (more pronounced for PPA), consistent with charge transfer from the cations. In addition, charge accumulation between the phosphonic group and surface halides suggests a partial covalent character.

When Pb atoms are exposed at the surface, reduced coordination enhances their cationic character. Upon passivation with PPA or CEPA, charge is transferred from undercoordinated Pb sites to oxygen atoms through the Pb–O bonding (Figure 6), leading to depletion of electron density around Pb and accumulation around the O atom. The intensity of the EDD isosurface correlates with the calculated *E*_int_, particularly when comparing d-2MAI and 2MAI: d-2MAI exhibits a more exergonic *E*_int_ (stronger interaction) and a more intense isosurface of the EDD. This correlation supports the conclusion that the higher stability of d-2MAI arises from more favorable intermolecular interactions than in 2MAI.

### 3.5 Electronic Structure Formation from Local Density of States

The LDOS profiles in Figure 7 provide element-resolved contributions for the adsorbed systems. Consistent with previous reports^31–33^ and with the 3D bulk electronic structure, the band edges arise mainly from the PbI_6_ octahedra: the valence-band maximum (VBM) is dominated by I *p* states with minor Pb *s* contributions, whereas the conduction-band minimum (CBM) is largely derived from Pb p states with smaller I p contributions. The dominance of the iodine character at the edge of the valence is consistent with the largely ionic nature of MAPbI_3_.

**Figure 7:**
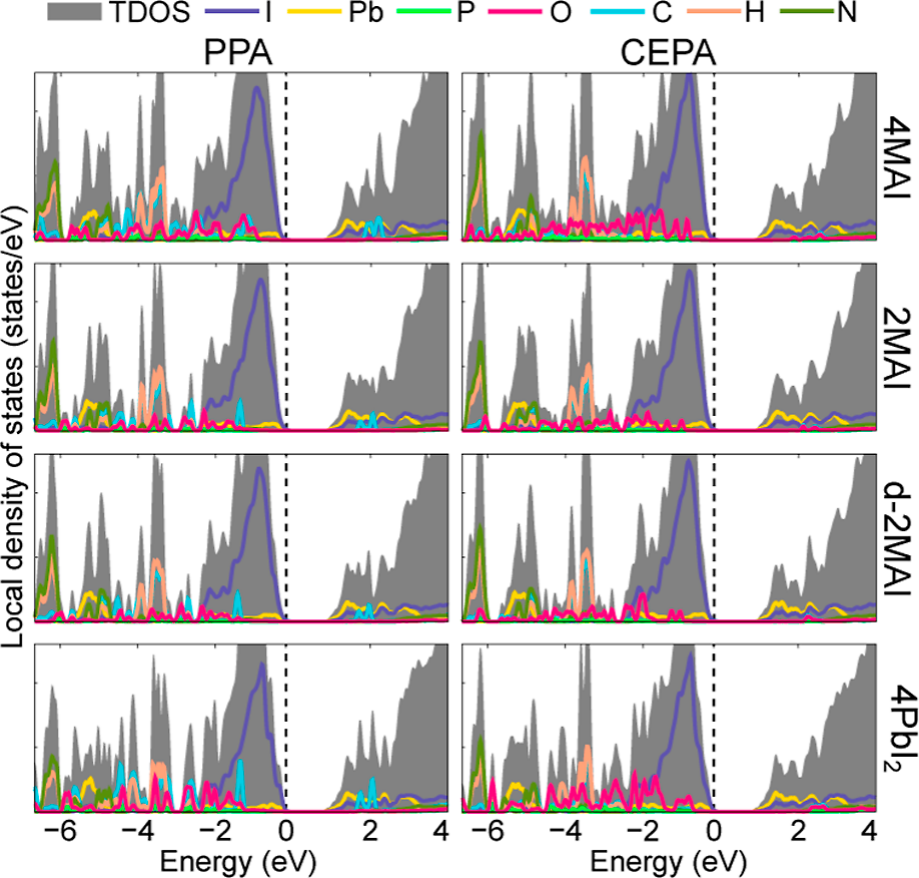
Local density of states for the PPA and CEPA on perovskite surface models: 4MAI, 2MAI, d-2MAI and 4PbI_2_, the valence band maximum was set at 0 eV (vertical dashed line).

Upon adsorption of PPA, additional carbon-derived states appear near the valence band, originating from the aromatic ring and being more pronounced for the fully Pb-exposed 4PbI_2_ surface. In CEPA, the CH_2_CH_2_COOH moiety does not introduce carbon states comparable in energy and symmetry to the aromatic ring of PPA’s, and its carbon contributions remain at lower energy. For both adsorbates, oxygen atoms in the phosphonic-acid group (R–PO­(OH)_2_) contribute to the valence region, with CEPA-derived states lying closer to the VBM. These mainly oxygen p states are energetically close to the PbI_6_ framework, indicating a possible hybridization with lead orbitals. In addition, oxygen states from the carboxylic group of CEPA’s (R–COOH) appear in the conduction band across the surface models, further enriching the electronic structure of the interface.

### 3.6 Work Function Modulation upon Surface Functionalization

Electron transport at the perovskite/electron–transport-layer interface is strongly influenced by the work function.^30^ Consistent with the definition Φ = *V*_es_(**r**_vac_) – *E*_Fermi_, we report Φ using the VBM-referenced upper-bound ionization-energy approximation for these semiconducting slabs, Φ ≈ *V*_es_(**r**_vac_) – *E*_VBM_ (see discussion above). To assess the effect of adsorption, we compute the passivation-induced change in work-function ΔΦ = Φ^acids+slab^ – Φ^slab^, with results summarized in Figure 8. CEPA systematically yields work functions that are higher than those of PPA on all surfaces. For 4MAI, CEPA increases Φ to 5.71 eV, whereas PPA yields 4.58 eV, consistent with a larger adsorption-induced electrostatic potential step (interfacial dipole) for CEPA.

**Figure 8:**
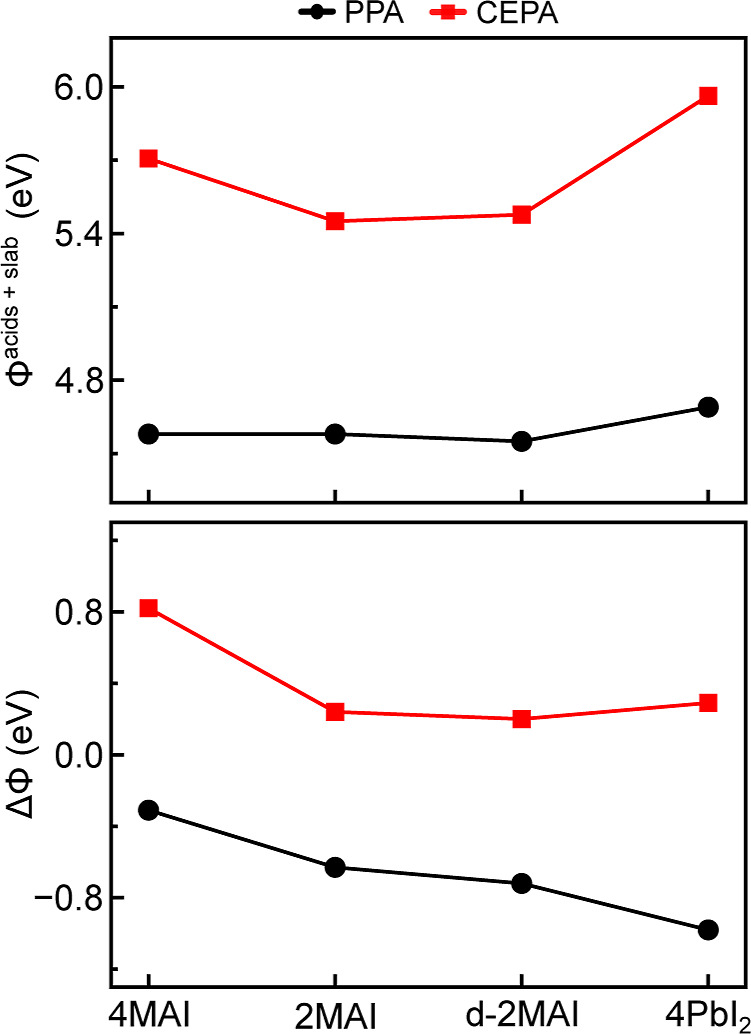
Top panel shows the VBM-referenced work function Φ (ionization-potential upper-bound approximation; see text), while the bottom panel displays the induced change ΔΦ for PPA and CEPA on perovskite surface models: 4MAI, 2MAI, d-2MAI, and 4PbI_2_.

The contrasting behaviors of PPA and CEPA highlight the key role of surface termination and adsorption geometry. PPA shows little dependence on the coverage of MAI, as indicated by the nearly identical Φ values for 2MAI and 4MAI. This suggests that PPA passivation is mainly governed by the availability of exposed Pb coordination sites rather than by the concentration or distribution of organic species. In contrast, CEPA depends more strongly on the coverage of MAI and the geometry of the adsorption-site. The complete coverage of MAI in 4MAI may enable a more cooperative alignment of the CEPA molecules, increasing the net surface dipole. Linear exposed-site configurations (e.g., 2MAI) may also allow more effective dipole–dipole alignment than diagonal arrangements such as d-2MAI. These results emphasize the need for precise interfacial engineering, particularly when using longer-chain passivating agents.

### 3.7 Analysis of the Lead–Oxygen Bonding

To improve our understanding of the nature and strength of chemical bonding in these systems, we performed crystal orbital Hamilton population (COHP) analysis^34^ using the local orbital basis suite toward electronic structure reconstruction (LOBSTER). The LOBSTER approach decomposes the band-structure energy into bonding, nonbonding, and antibonding contributions between selected atomic pairs by projecting onto a localized atomic basis. The pbsVaspFit2015 projection basis was used for all calculations.^35,36^ The COHP for a pair of neighboring atoms (μ, ν) at energy *E* is defined as

6 $${COHP}_{\mu \mathrm{T⃗},\nu {\mathrm{T⃗}}^{\prime }}\left(E\right)=H_{\mu \mathrm{T⃗},\nu {\mathrm{T⃗}}^{\prime }}\sum_{j,\mathrm{k⃗}}f_{j}\left(\mathrm{k⃗}\right)C_{\mu \mathrm{T⃗},j}^{*}\left(\mathrm{k⃗}\right)\times C_{\nu {\mathrm{T⃗}}^{\prime },j}\left(\mathrm{k⃗}\right)\delta \left(\epsilon_{j}\left(\mathrm{k⃗}\right)-E\right)$$

where *H*_μν_ is the Hamiltonian matrix element between atomic orbitals μ and ν; *C*_μ,*j*_(**k**) is the eigenvector coefficient of atomic orbital μ for band *j* at momentum **k**; ϵ_*j*_(**k**) is the band energy; *f*_*j*_(**k**) is the occupation number; and *T⃗* and ${\mathrm{T⃗}}^{\prime }$ denote the lattice vectors that specify the unit cells in which orbitals μ and ν are located, respectively. Thus, μ*T⃗* and $\nu {\mathrm{T⃗}}^{\prime }$ identify atomic orbitals centered on different (or the same) unit cells within the periodic crystal structure.^35,37,38^

The results are shown in Figure 9. The *x*-axis reports negative COHP values (−COHP), where the values to the right of 0.0 indicate bonding contributions and the values to the left indicate antibonding contributions. The *y*-axis shows the energy relative to the Fermi level (*E* – *E*_Fermi_), with *E*_Fermi_ set to 0.0 eV.

**Figure 9:**
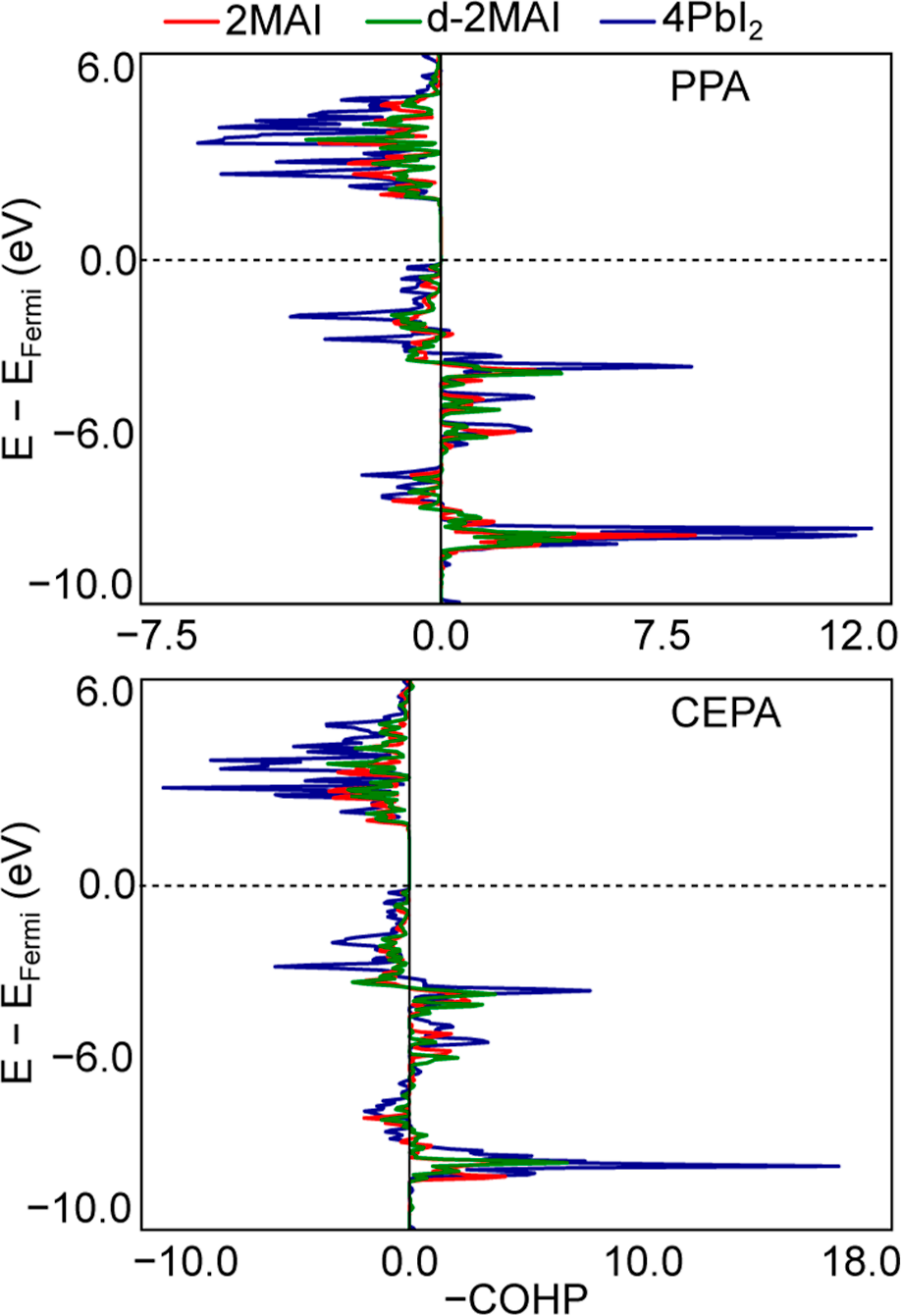
Crystal orbital Hamilton population analysis of PPA and CEPA adsorption on perovskite surface models: 4MAI, 2MAI, d-2MAI and 4PbI_2_, focusing on Pb–O bond character.

Direct comparison of the COHP profiles for PPA and CEPA shows that CEPA yields bonding contributions extending to more negative −COHP values in certain energy regions, reflected by a broader distribution of bonding states along the *x*-axis. However, peak −COHP amplitudes alone do not quantify the bond strength. A more rigorous metric is the integrated COHP (ICOHP), defined as the energy integral of the COHP up to the Fermi level; more negative ICOHP values indicate stronger covalent interactions.^35,37,38^ The ICOHP results indicate that the overall bond strength Pb–O is similar for PPA and CEPA and is governed mainly by surface termination rather than by the specific adsorbate. The absence of significant occupied antibonding states for both acids further supports the formation of strong, thermodynamically favorable Pb–O bonds upon adsorption, consistent with the adsorption-energy trends (*E*_ad_).

We further quantified the bonds strengths Pb–O for the adsorption of PPA and CEPA in 4MAI, 2MAI, d-2MAI, and 4PbI_2_ using ICOHP. For PPA, the ICOHP values for 4PbI_2_, 2MAI, and d-2MAI are −1.93 eV, −1.40 eV, and −1.68 eV, respectively. For CEPA, the corresponding values are −1.94 eV, −1.38 eV, and −1.53 eV. These results show that 4PbI_2_ forms the strongest and most thermodynamically stable Pb–O bonds, consistent with the interpretation that more negative ICOHP values indicate stronger covalent interactions.

By correlating the computed ICOHP values with the corresponding Pb–O bond distances (*d*_av_^Pb–O^), we identified a relationship between interatomic separation and bond stability for acids adsorbed on perovskite surfaces featuring undercoordinated lead centers. The correlation between *d*_av_^Pb–O^ and ICOHP is shown in Figure 10.

**Figure 10:**
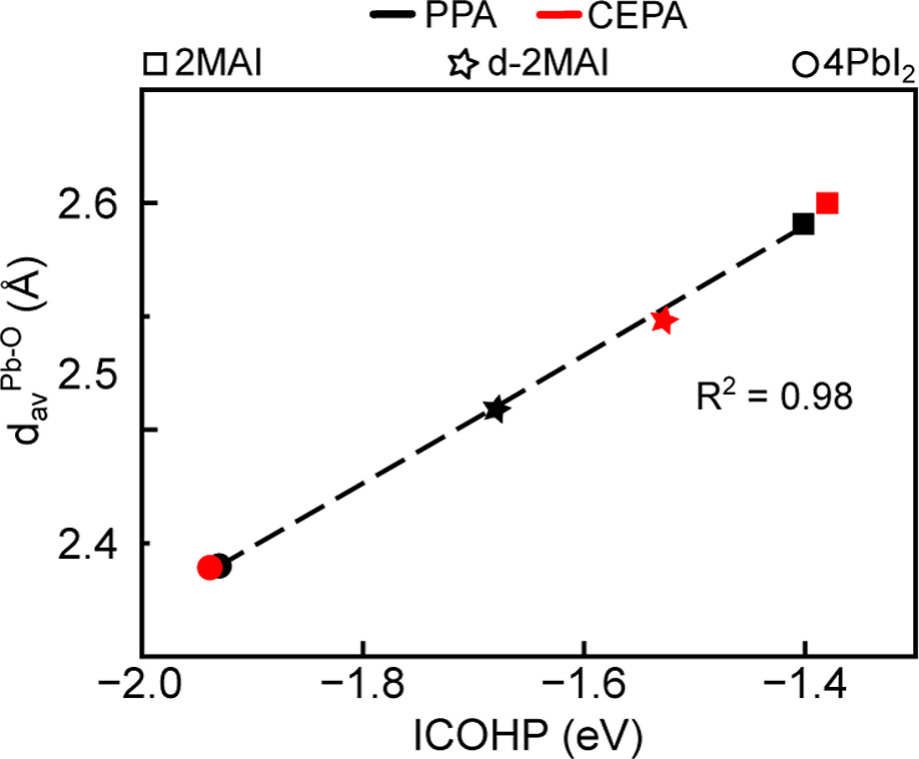
Linear correlation between the Pb–O bond distances (*d*_av_^Pb–O^) and the integrated crystal orbital Hamilton population values. Different data points represent the perovskites models: 2MAI (squares), d-2MAI (stars), and 4PbI_2_ (circles). A dashed line highlights the observed linear trend.

As anticipated, a strong linear correlation (*R* ^2^ = 0.98) was observed in all investigated systems, demonstrating that shorter distances Pb–O are associated with more negative ICOHP values and, consequently, with stronger bonding interactions. Among the three surfaces, 4PbI_2_ displays the shortest Pb–O bond lengths and the most negative ICOHP values for both adsorbates, indicating enhanced orbital overlap and increased electronic stabilization at the interface, this behavior can be further understood from the LDOS associated with the Pb–O bond, as shown in Figure 11. This trend underscores the stabilizing role of acid passivation: the most stable systems are characterized by smaller values of *d*_av_^Pb–O^ and a more pronounced covalent character of the Pb–O bond.

**Figure 11:**
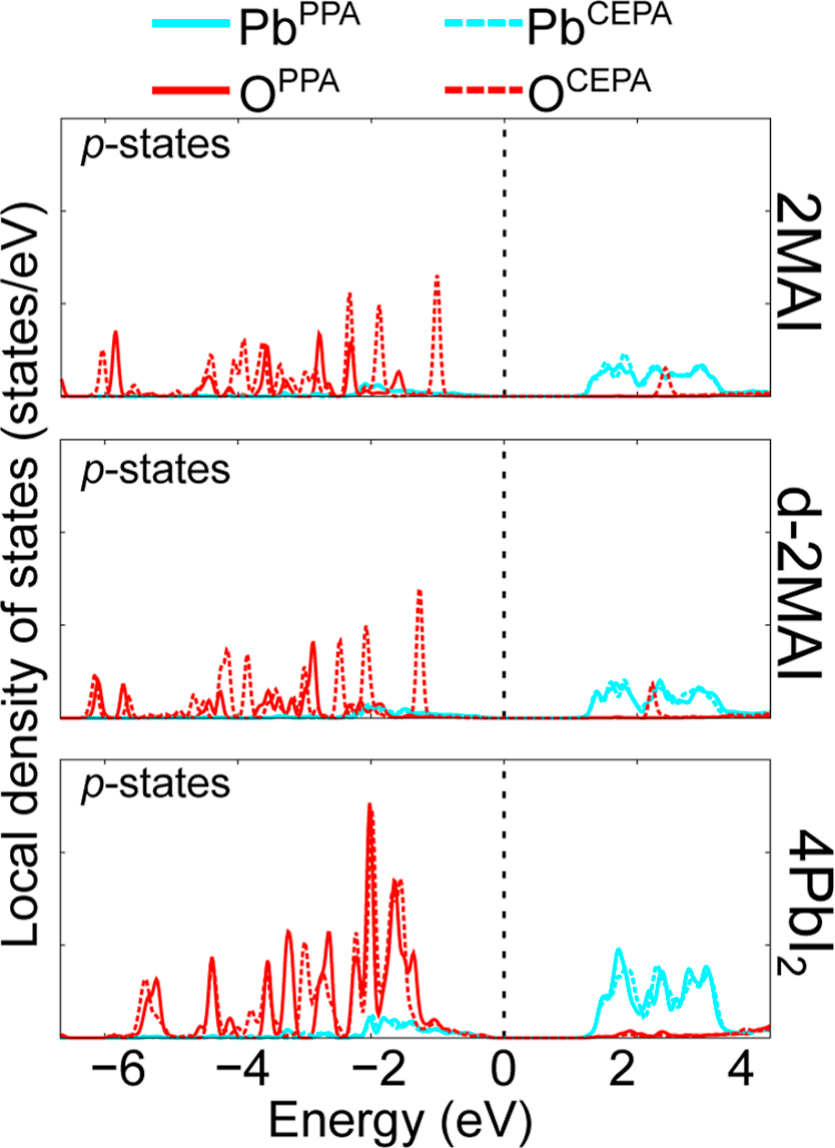
LDOS highlighting Pb and O p-state contributions at Pb–O bonds for various perovskite surface models: 4MAI, 2MAI, d-2MAI, and 4PbI_2_. Calculations were performed using PPA and CEPA acids. The valence band maximum is referenced at 0 eV (vertical dashed line), with a displayed energy range from 0 to 15 eV.

### 3.8 Insights from Phosphonic Acids Passivation

#### 3.8.1 Energetic Stability

Passivating perovskite surfaces with undercoordinated Pb ions through phosphonic-acid adsorption is thermodynamically favorable, as shown by consistently negative adsorption and interaction energies. The stronger binding of CEPA relative to that of PPA highlights the influence of functional-group chemistry on passivation efficiency. The carboxyl group (−COOH) in CEPA provides additional hydrogen-bonding interactions that strengthen surface anchoring beyond Pb–O coordination. However, under photovoltaic operating conditions, the higher hydrophilicity of CEPA can reduce the stability of the device by facilitating interactions with H_2_O.

#### 3.8.2 Charge Redistribution

Effective-charge analysis pinpoints the Pb–O–P group as the main site of electronic stabilization: oxygen atoms gain charge and Pb atoms become more positive. Regardless of acid type, the effective charges of I and MA remain nearly unchanged. The phosphorus charges indicate that CEPA is more polarizing than PPA, consistent with a stronger donor–acceptor character of the Pb–O bond. Electron density difference maps depict charge redistribution at the interface. For MAI-rich surfaces, the accumulation of charge at the acid–halide contact indicates an increased covalent character, whereas Pb-exposed surfaces show charge transfer from the undercoordinated Pb sites to acid oxygens. The intensity of these features scales with the interaction energy, suggesting that experimental probes of the interfacial bond strength could indicate passivation efficiency.

#### 3.8.3 Electronic Structure and Work Function Modulation

LDOS calculations show that adsorption modifies the band edges mainly through oxygen- and carbon-derived states. PPA introduces aromatic states near the valence band, whereas CEPA shifts oxygen-derived states closer to the VBM and CBM, indicating stronger hybridization with Pb orbitals. This is consistent with experimentally observed trap suppression in CEPA-treated systems.^12^ The larger increase in work-function induced by CEPA relative to PPA highlights the role of molecular dipoles. Long-chain acids such as CEPA can promote cooperative dipole alignment on perovskite surfaces, producing larger vacuum-level shifts. Aalbers et al.^12^ reported a higher work function for CEPA-functionalized substrates than for PPA, consistent with this picture.

#### 3.8.4 Bonding Strength

The COHP and ICOHP analyses show that the Pb–O bond strength scales with the Pb–O distance. This correlation links local bonding motifs to interfacial energetics and provides a basis for molecular screening.

## 4 Conclusions

In this study, we used DFT calculations to investigate the interaction of PPA and CEPA with various MAPbI_3_ surface terminations. By combining adsorption-energy calculations with charge-density and electronic structure analyzes, we established a microscopic framework that links molecular functionality with changes in the stability and electronic structure of hybrid perovskite surfaces.

Both acids adsorb favorably on all examined terminations, with CEPA consistently binding more strongly than PPA, primarily because its carboxyl group enables additional hydrogen-bonding interactions. The adsorption strength increases with the degree of exposure of the surface Pb, and the 4PbI_2_ and d– 2MAI terminations show the most favorable binding. Charge-redistribution and local-density-of-states analyses indicate that Pb–O coordination is the dominant anchoring motif, while cooperative interactions further enhance passivation. In particular, CEPA induces pronounced interfacial dipole shifts, raising the work function to values exceeding 5.9 eV and modulating interfacial energy-level alignment.

Overall, these findings support phosphonic acids—particularly CEPA—as effective molecular passivators that can enhance structural stability, electronic passivation, and interfacial energetics. By linking molecular-level chemistry to interface-relevant properties, this work provides a framework for rational interface engineering in halide perovskites and suggests pathways toward more stable and efficient perovskite solar cells.

## Supplementary Material



## Notes

(i) Additional methodological details of the DFT setup, including the specific PAW data sets employed (projector identifiers, *Z*_val_, and ENMAX); (ii) stress-tensor-based convergence tests for the MAPbI_3_ slab models (pressure, residual forces, Δ*E*_tot_, and Δ*V* across successive relaxation cycles for 4*AX*, 2*AX*, 2*AX*_d_, and 4PbI_2_); (iii) optimized adsorption geometries of PPA- and CEPA-passivated slabs; and (iv) complementary work-function results, reporting *V*_es_(**r**_vac_), VBM, and Φ for each termination/passivator combination (PDF)

## Abbreviations

DFT: density functional theory
PBE: Perdew–Burke–Ernzerhof
VASP: Vienna Ab initio Simulation Package
