# Supplementary material for: Enhancing Surface Termination and Stability of Hybrid Halide Perovskites via Phosphonic Acid Passivation
Source: ACS Omega. 2026 Apr 28;11(18):26456–67. doi: 10.1021/acsomega.5c12245 (PMC13176983; doi:10.1021/acsomega.5c12245)
Supplement: Supplementary file 1 [file ao5c12245_si_001.pdf]

Electronic Supporting Information: Enhancing Surface Termination and Stability of Hybrid Halide Perovskites via Phosphonic Acid Passivation

Israel C. Ribeiro,[*,†] Iván Ornelas-Cruz,[*,†] Felipe D. Picoli,[*,‡] Luiz N. Oliveira,[*,‡] Matheus P. Lima,[*,¶] Ana Flávia Nogueira,[*,§] and Juarez L. F. Da Silva[*,†]

†*São Carlos Institute of Chemistry, University of São Paulo, Av. Trabalhador São-Carlense* 400, 13560-970, *São Carlos, SP, Brazil*

‡*São Carlos Institute of Physics, University of São Paulo, Av. Trabalhador São-Carlense* 400, 13560-970, *São Carlos, SP, Brazil*

¶*Department of Physics, Federal University of São Carlos,* 13565-905, *São Carlos, SP, Brazil*

§*Laboratório de Nanotecnologia e Energia Solar, Chemistry Institute, University of Campinas,* 13083-970, *Campinas, SP, Brazil*

E-mail: israelribeiroc7@gmail.com; iornelas.ipn@gmail.com; felipedonipicoli@gmail.com; luizno@usp.br; mplima@df.ufscar.br; anafla@unicamp.br; juarez_dasilva@iqsc.usp.br

Contents

S-1 Introduction **S-2**

S-2 Additional Details on Selected PAW Projectors **S-2**

S-3 Convergence Tests for Perovskite Slab **S-3**

S-4 Phosphoryl-based Molecules Passivation **S-7**

S-4.1 Slab Passivation with PPA and CEPA S-7

S-4.2 Work Function S-9

References **S-10**

S-1 INTRODUCTION

This supplementary electronic supporting information file presents additional methodological details of the density functional theory simulations, together with complementary computational results, intended to facilitate comprehension and reproducibility for researchers who are new to this field.

S-2 ADDITIONAL DETAILS ON SELECTED PAW PROJECTORS

The projector augmented wave (PAW) method,[1,2], as implemented in the Vienna Ab initio Simulation Package (VASP),[3,4] was used for all density functional theory calculations.[5,6] Within VASP, only a restricted set of PAW data sets are available for each chemical element. Consequently, in the present work we systematically adopted the most up-to-date PAW datasets featuring the GW extension,[7] which are summarized in Table S-1. These datasets are specifically optimized to provide an improved description of unoccupied electronic states, a feature that is essential for an accurate evaluation of optical properties,[8] among other calculations of excited-state and response-function.

Table S-1. Technical specifications of the selected PAW projectors, including the chemical species, projector identifier (project name), maximum recommended plane-wave cutoff energy (`ENMAX`, in eV), number of valence electrons (Z_{val}), and corresponding valence electronic configurations.

Atoms	PAW Projectors	`ENMAX`	Z_{val}	Valence
H	H_GW 21Apr2008	300.000	1	$1s^1$
C	C_GW_new 28Sep2005	413.992	4	$2s^2 2p^2$
N	N_GW_new 19Mar2012	420.902	5	$2s^2 2p^3$
P	P_GW 19Mar2012	255.040	5	$3s^2 3p^3$
O	O_GW_new 19Mar2012	434.431	6	$2s^2 2p^4$
I	I_GW 12MAr2012	175.647	7	$5s^2 5p^5$
Pb	Pb_d_GW 14Apr2014	237.809	16	$5d^{10} 5s^2 6s^2 6p^2$

S-3 CONVERGENCE TESTS FOR PEROVSKITE SLAB

The investigation started with the selection of four distinct surfaces for passivation using phosphoryl-functionalized molecular species. Each surface consists of a two-dimensional $MAPbI_3$ thin film with a thickness of 26 Å, consisting of four stacked layers of PbI_6 octahedra. To elucidate the influence of coordination interactions between surface lead atoms and phosphoryl groups, we deliberately introduced halide vacancies at the topmost surface of each film. The reference model, designated as 4 PbI_2, corresponds to a configuration in which all iodine atoms on the top surface are removed, resulting in complete exposure of the surface lead atoms.

To evaluate the influence of this coordination on the stability of the system, we constructed a series of additional control models. Among these, model $4AX$ preserves all iodine atoms and thus contains no vacancies. In addition to these two limiting cases, we investigated two intermediate models in which half of the iodine atoms were removed, leading to a partial exposure of lead atoms at the top surface. Owing to the intrinsic symmetry of the surface, these models allow the exposed lead atoms to be oriented either parallel or perpendicular to the a_0 axis. The configuration with lead atoms aligned parallel to a_0 is denoted as $2AX$, whereas the configuration in which the exposed lead atoms are oriented perpendicular to a_0 is referred to as $2AX_d$. All models are illustrated in Figure S-1.

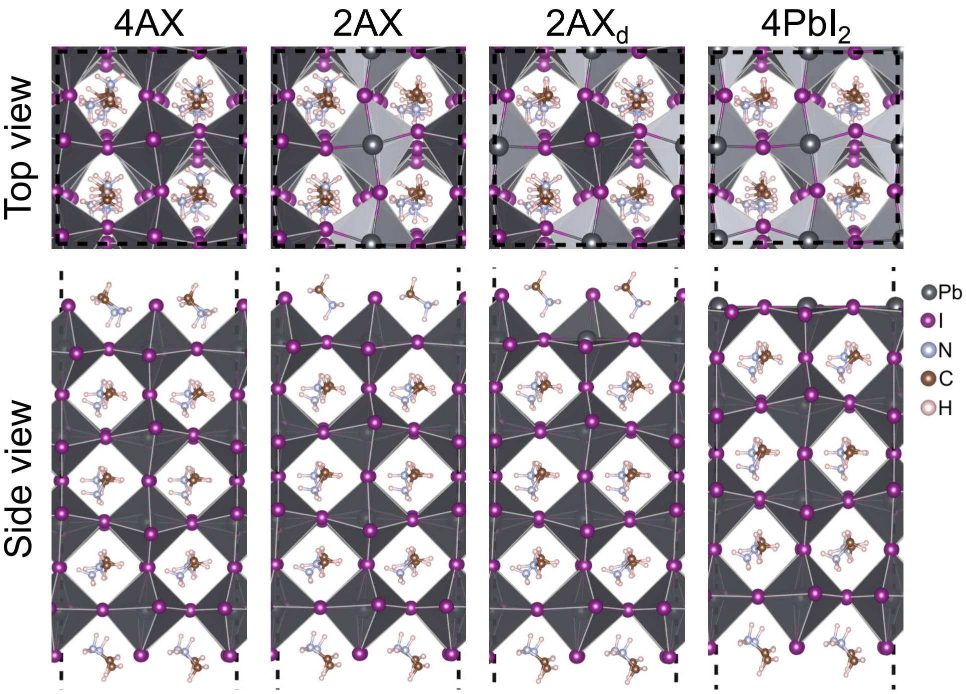

Figure S-1. Top and side views of the surface models corresponding to three distinct configurations of the $MAPbI_3$ thin film. The models comprise: (*i*) a fully exposed lead-terminated surface ($4\,PbI_2$); (*ii*) a surface without iodine vacancies ($4AX$); and (*iii*) two intermediate configurations in which half of the iodine atoms are removed, denoted as $2AX$ (exposing lead atoms parallel to the a_0 axis) and $2AX_d$ (exposing lead atoms perpendicular to the a_0 axis).

For each surface model, systematic convergence tests were performed based on stress-tensor calculations. The structural optimization was carried out through sequential ionic relaxation cycles, each consisting of approximately 20 ionic steps, allowing for a gradual and controlled assessment of convergence. On average, six such cycles were required per slab, with some cases necessitating up to eight cycles to attain full optimization. This protocol was applied consistently to all four slab models ($4AX$, $2AX$, $2AX_d$, and $4\,PbI_2$), thus ensuring structurally robust and mechanically stable configurations. Subsequently, we examine the evolution of total energy, cell volume, pressure, and atomic forces as key observables to quantify and validate the convergence of the stress tensor in the optimization runs for each slab.

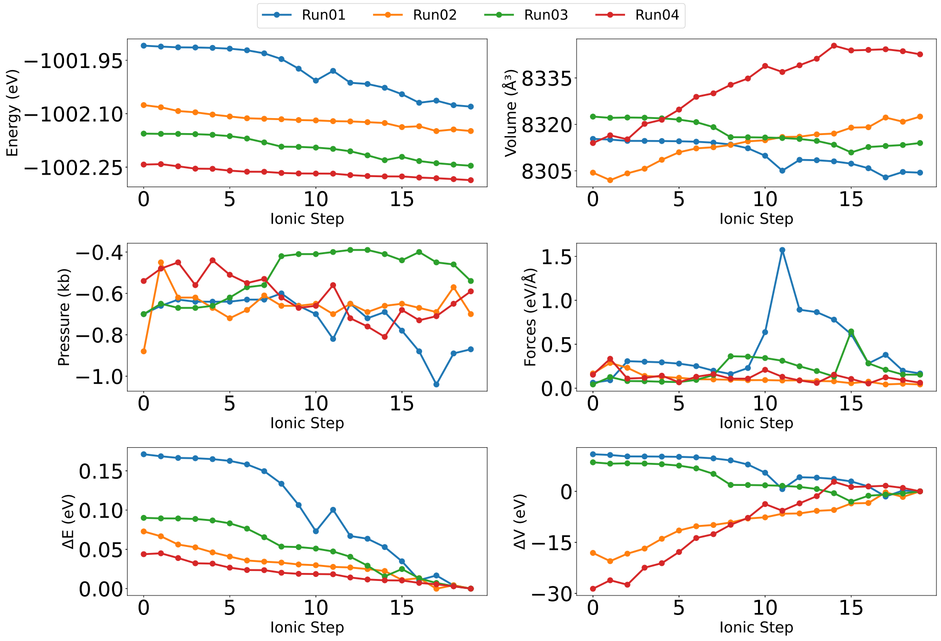

Figure S-2. Convergence criteria for the $4AX$ model across the optimization runs, including pressure, atomic forces, relative total energy (ΔE_{tot}), and relative volume (ΔV).

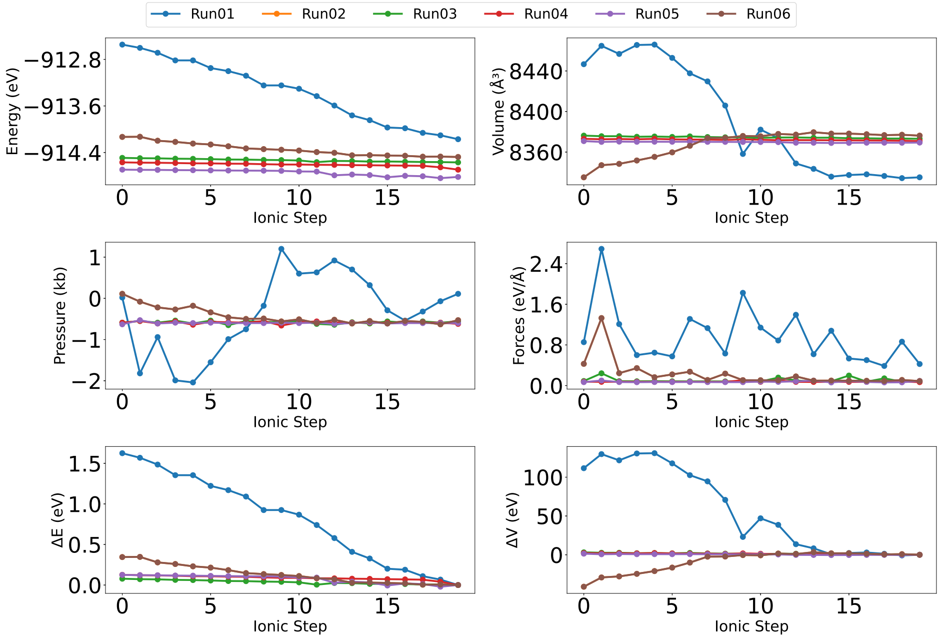

Figure S-3. Convergence criteria for the $2AX$ model across successive optimization runs, encompassing pressure, atomic forces, relative total energy (ΔE_{tot}), and relative volume (ΔV).

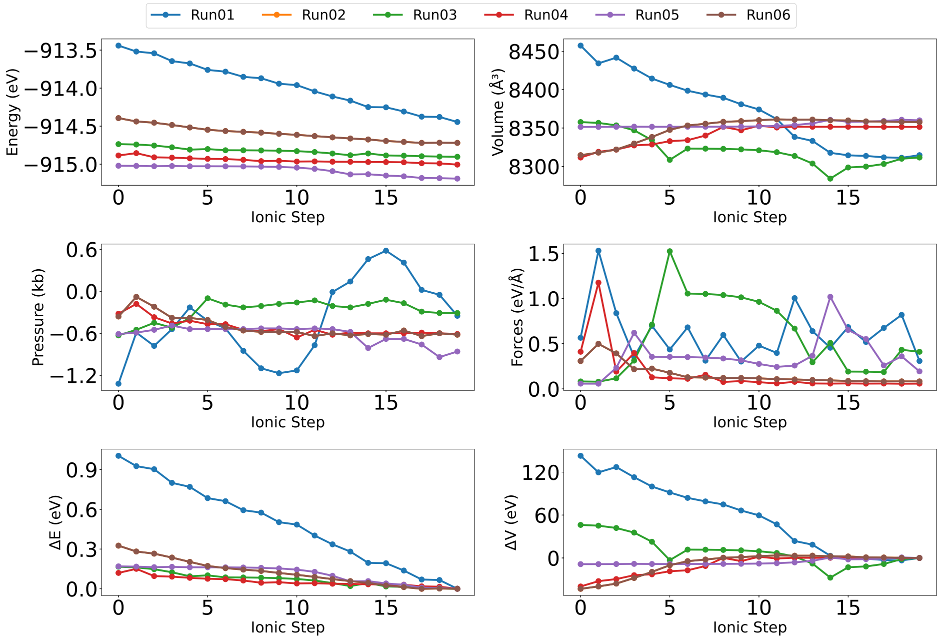

Figure S-4. Convergence parameters for the $2AX_d$ model over multiple optimization runs, including pressure, atomic forces, relative total energy (ΔE_{tot}), and relative volume (ΔV).

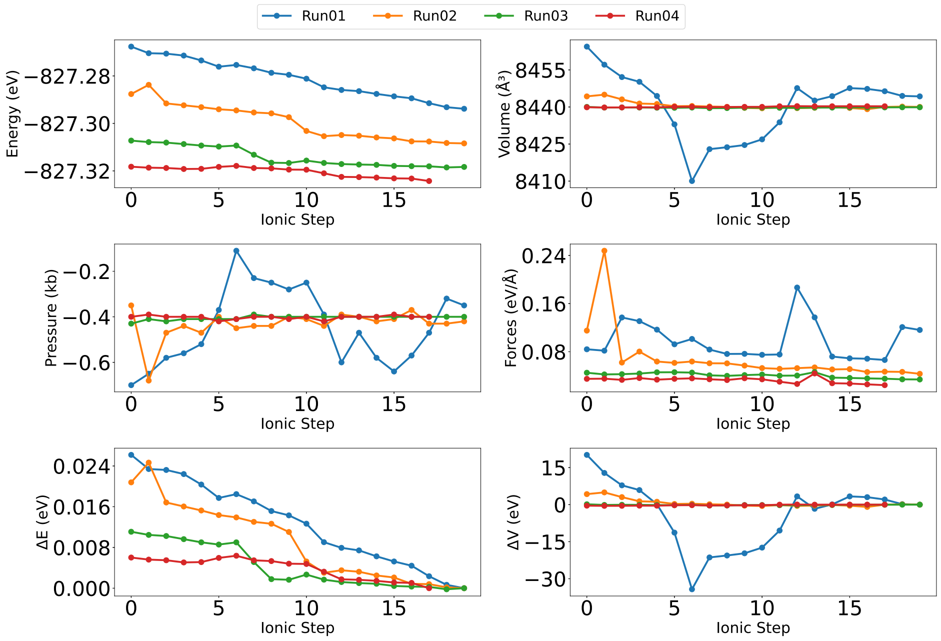

Figure S-5. Convergence criteria for the $4\,PbI_2$ model over successive geometry optimization cycles, including pressure, atomic forces, relative total energy (ΔE_{tot}), and relative volume (ΔV).

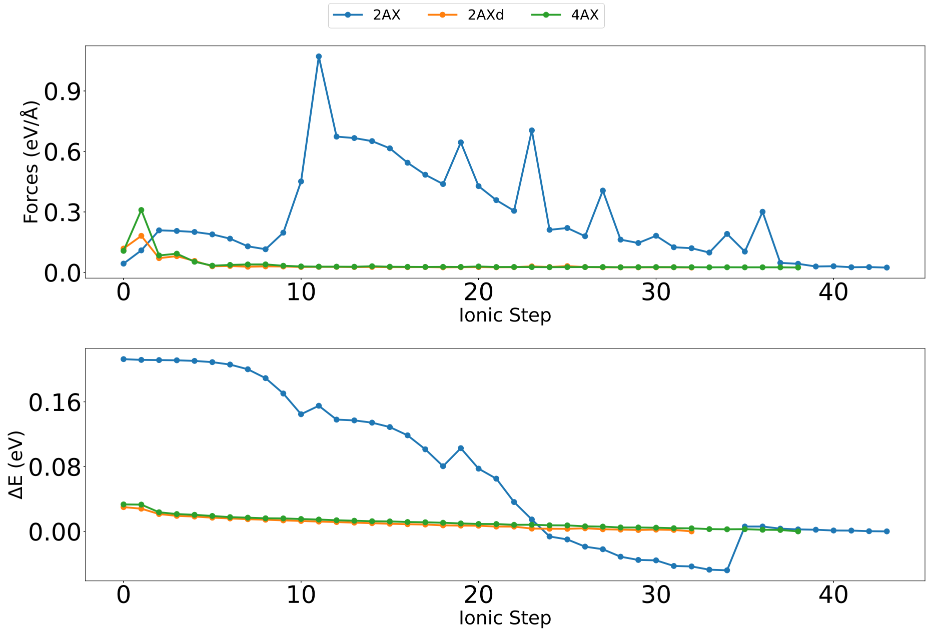

Figure S-6. Convergence parameters for the $2AX$, $2AX_d$, and $4AX$ models during the final optimization iterations, specifically the residual forces and the relative total energy (ΔE_{tot}).

S-4 PHOSPHORYL-BASED MOLECULES PASSIVATION

S-4.1 Slab Passivation with PPA and CEPA

Upon optimization and determination of the equilibrium volumes for each slab, following the procedure outlined in V_0, we proceeded to the passivation step using phosphoryl-based molecules. For this purpose, two molecular species were selected: phenylphosphonic acid and 2-carboxyethylphosphonic acid. The atomic configurations corresponding to the passivated surfaces obtained with each molecule are shown in Figures S-7 and S-8, respectively.

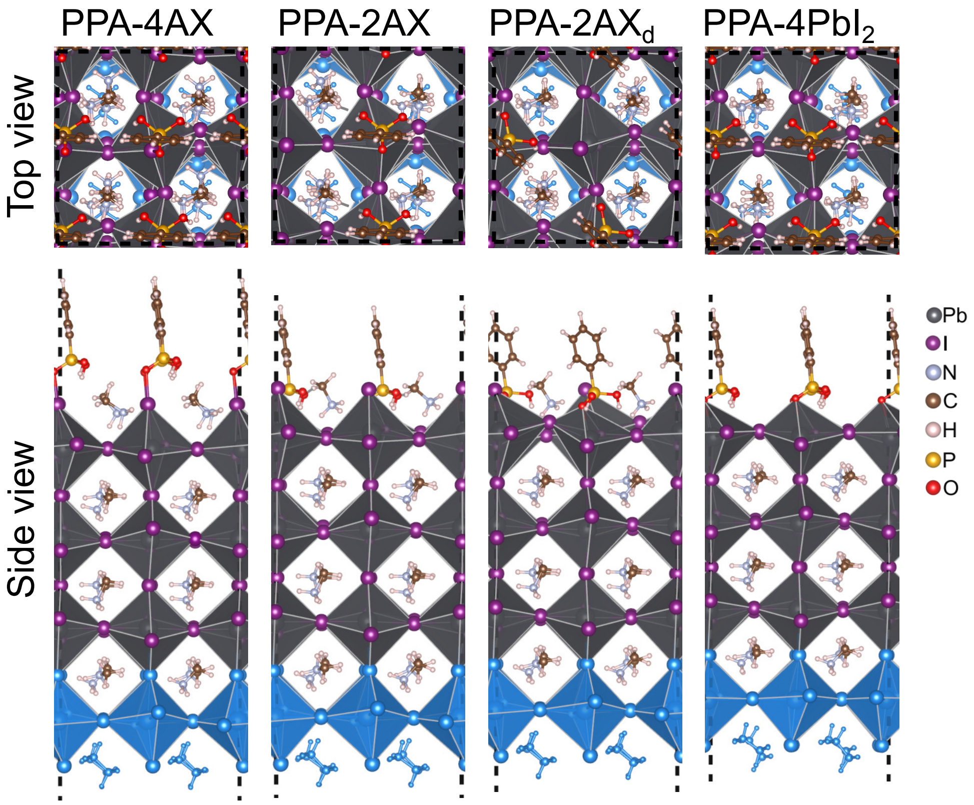

Figure S-7. Top and side views of PPA passivation on the $4AX$, $2AX$, $2AX_d$, and $4\,PbI_2$ perovskite surfaces. Blue-colored atoms denote the constrained bottom surface, while the dashed line delineates the boundaries of the unit cell.

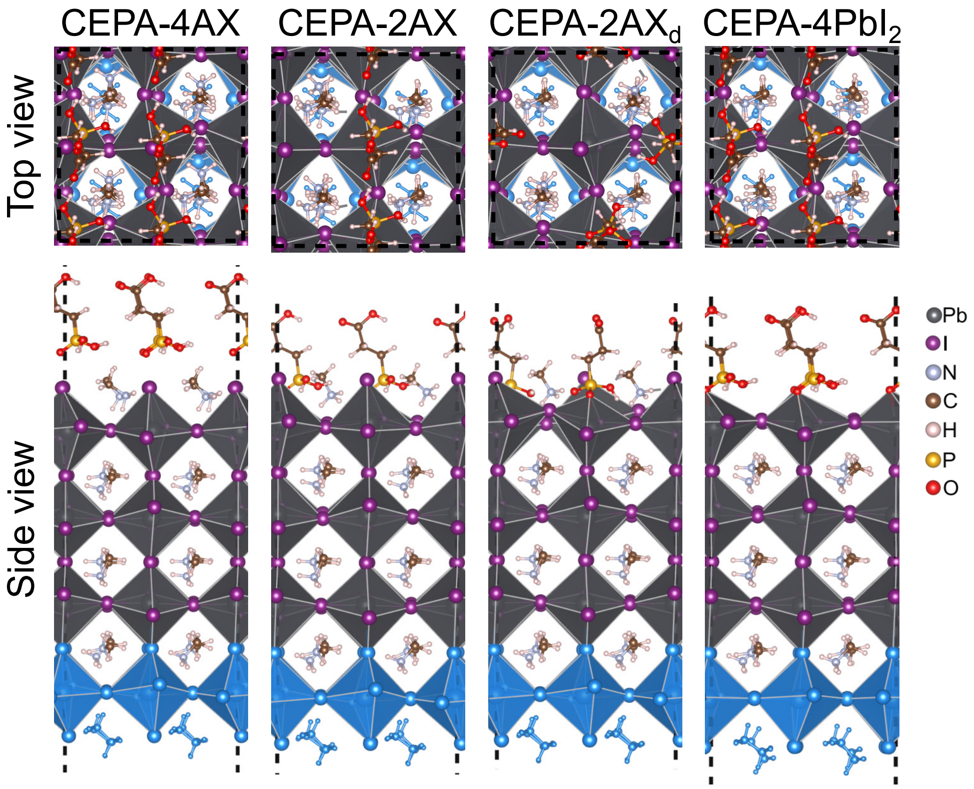

Figure S-8. Top and side views of CEPA passivation on the $4AX$, $2AX$, $2AX_d$, and $4\,PbI_2$ perovskite surfaces. Blue atoms denote the fixed bottom surface, while the dashed line delineates the unit cell.

S-4.2 Work Function

In this work, the work function (Φ) is defined as the energy difference between the vacuum level (E_{vac}) and the Valence Band Maximum (VBM). For intrinsic halide perovskites, the Fermi level (E_F) typically resides within the bandgap. Using the VBM as the reference level for Φ provides a more stable physical threshold for analyzing energy level alignment and interfacial charge transfer in optoelectronic devices. This approach directly captures the influence of surface dipoles and stoichiometry on the electrostatic potential landscape.

Table S-2. Work function (Φ), vacuum energy level ($V_{es}(\mathbf{r}_{vac})$), and valence band maximum (VBM) for perovskite systems based on PPA and CEPA.

Acid	Surface	$V_{es}(\mathbf{r}_{vac})$ (eV)	VBM (eV)	Φ (eV)
PPA	4 AX	3.46	−1.12	4.58
	2 AX	3.09	−1.49	4.58
	2 AXd	3.08	−1.47	4.55
	4 PbI_2	3.38	−1.31	4.69
CEPA	4 AX	4.33	−1.38	5.71
	2 AX	3.72	−1.74	5.45
	2 AXd	3.74	−1.74	5.48
	4 PbI_2	4.24	−1.73	5.96

References

1 Blochl, P. E. Projector Augmented-wave Method. *Phys. Rev. B* **1994**, *50*, 17953–17979, DOI: 10.1103/PhysRevB.50.17953.

2 Kresse, G.; Joubert, D. From Ultrasoft Pseudopotentials to the Projector Augmented-wave Method. *Phys. Rev. B* **1999**, *59*, 1758–1775, DOI: 10.1103/PhysRevB.59.1758.

3 Kresse, G.; Hafner, J. *Ab initio* Molecular Dynamics for Open-shell Transition Metals. *Phys. Rev. B* **1993**, *48*, 13115–13118, DOI: 10.1103/physrevb.48.13115.

4 Kresse, G.; Furthmüller, J. Efficient Iterative Schemes for *Ab initio* Total-energy Calculations Using a Plane-wave Basis set. *Phys. Rev. B* **1996**, *54*, 11169–11186, DOI: 10.1103/physrevb.54.11169.

5 Hohenberg, P.; Kohn, W. Inhomogeneous Electron Gas. *Phys. Rev.* **1964**, *136*, B864–B871, DOI: 10.1103/PhysRev.136.B864.

6 Kohn, W.; Sham, L. J. Self-consistent Equations Including Exchange and Correlation Effects. *Phys. Rev.* **1965**, *140*, A1133–A1138, DOI: 10.1103/PhysRev.140.A1133.

7 Shishkin, M.; Kresse, G. Self-consistentGWcalculations for semiconductors and insulators. *Physical Review B* **2007**, *75*, DOI: 10.1103/physrevb.75.235102.

8 Adolph, B.; Furthmüller, J.; Bechstedt, F. Optical Properties of Semiconductors Using Projector-Augmented Waves. *Phys. Rev. B* **2001**, *63*, 125108, DOI: 10.1103/physrevb.63.125108.
